# Space–time clustering patterns in childhood leukaemia support a role for infection

**DOI:** 10.1054/bjoc.1999.1072

**Published:** 2000-04-03

**Authors:** J M Birch, F E Alexander, V Blair, O B Eden, G M Taylor, R J Q McNally

**Affiliations:** CRC Paediatric & Familial Cancer Research Group, Royal Manchester Children's Hospital, Stancliffe, Hospital Road, Manchester, M27 4HA; Department of Public Health Sciences, The University of Edinburgh Medical School, Teviot Place, Edinburgh, EH8 9AG, UK; Academic Unit of Paediatric Oncology, Royal Manchester Children's Hospital and Christie Hospital Trusts, Hospital Road, Manchester, M27 4HA, UK; Immunogenetics Laboratory, Department of Medical Genetics, St Mary's Hospital, Manchester, M13 0JH, UK

**Keywords:** clustering, childhood leukaemia, infections

## Abstract

Previous studies of space–time clustering in childhood leukaemia have produced equivocal and inconsistent results. To address this issue we have used Manchester Children's Tumour Registry leukaemia data in space–time clustering analyses. Knox tests for space–time interactions between cases were applied with fixed thresholds of close in space, < 5 km and close in time < 1 year apart. Addresses at birth as well as diagnosis were utilized. Tests were repeated replacing geographical distance with distance to the Nth nearest neighbour. N was chosen such that the mean distance was 5 km. Data were also examined by a second order procedure based on K-functions. All methods showed highly significant evidence of space–time clustering based on place of birth and time of diagnosis, particularly for all leukaemias aged 0–14 and 0–4 years, and acute lymphoblastic leukaemia (ALL) 0–4 years. Some results based on location at diagnosis were significant but mainly gave larger *P* -values. The results are consistent with an infectious hypothesis. Furthermore, we found an excess of male cases over females involved in space–time pairs. We suggest this may be related to genetic differences in susceptibility to infection between males and females. These findings provide the basis for future studies to identify possible infectious agents. © 2000 Cancer Research Campaign


*British Journal of Cancer* (2000) **82**(9), 1571–1576
© 2000 Cancer Research Campaign
DOI: 10.1054/ bjoc.1999.1072, available online at http://www.idealibrary.com on IDEAL®

### Space–time clustering patterns in childhood leukaemia support a role for infection

**JM Birch[1], FE Alexander[2], V Blair[1], OB Eden[3], GM Taylor[4] and RJQ McNally[1]**

[1]CRC Paediatric & Familial Cancer Research Group, Royal Manchester Children's Hospital, Stancliffe, Hospital Road, Manchester M27 4HA; [2]Department of Public Health Sciences, The University of Edinburgh Medical School, Teviot Place, Edinburgh EH8 9AG, UK; [3]Academic Unit of Paediatric Oncology, Royal Manchester Children's Hospital, and Christie Hospital Trusts, Hospital Road, Manchester M27 4HA, UK; [4]Immunogenetics Laboratory, Department of Medical Genetics, St Mary's Hospital, Manchester M13 0JH, UK

**Summary** Previous studies of space–time clustering in childhood leukaemia have produced equivocal and inconsistent results. To address this issue we have used Manchester Children's Tumour Registry leukaemia data in space–time clustering analyses. Knox tests for space–time interactions between cases were applied with fixed thresholds of close in space, < 5 km and close in time < 1 year apart. Addresses at birth as well as diagnosis were utilized. Tests were repeated replacing geographical distance with distance to the Nth nearest neighbour. N was chosen such that the mean distance was 5 km. Data were also examined by a second order procedure based on K-functions. All methods showed highly significant evidence of space–time clustering based on place of birth and time of diagnosis, particularly for all leukaemias aged 0–14 and 0–4 years, and acute lymphoblastic leukaemia (ALL) 0–4 years. Some results based on location at diagnosis were significant but mainly gave larger *P*-values. The results are consistent with an infectious hypothesis. Furthermore, we found an excess of male cases over females involved in space–time pairs. We suggest this may be related to genetic differences in susceptibility to infection between males and females. These findings provide the basis for future studies to identify possible infectious agents. © 2000 Cancer Research Campaign

**Keywords**: clustering; childhood leukaemia; infections

Three hypotheses have been proposed to explain the role that infection(s) may have in the aetiology of childhood leukaemia. Kinlen has suggested that situations of unusual population mixing would result in an increased level of contacts between susceptible and infected individuals (Kinlen, 1988). This idea has been tested in a number of relevant populations and the findings have been uniformly consistent with the hypothesis (Kinlen, 1995). Greaves proposes that the common subtype of acute lymphoblastic leukaemia [c-ALL] arises as a result of two independent mutations; the first occurring in utero (Wiemels et al, 1999) or shortly after birth creates a preleukaemic clone of cells, with a second arising after an average period of 3 years precipitating the onset of disease. It is suggested that common infections serve as a promoter of the second mutation. Delay in the normal pattern of exposure of the immune system to infection would increase the pool of susceptible cells and hence the risk of the second critical mutation (Greaves, 1988). An alternative hypothesis explaining the childhood peak of ALL in populations of children in socio-economically developed communities attributes it to an in utero exposure to infection (Smith, 1997). We have examined acute leukaemia data from the Manchester Children's Tumour Registry (MCTR) for evidence of space–time clustering. The aims were: (1) to test predictions of space–time clustering patterns which might arise as a result of mechanisms proposed by Kinlen (1988) and Greaves (1988); (2) to attempt to distinguish between hypotheses relating to time of onset or to prenatal events by utilizing locations and dates of birth as well as at diagnosis as proposed by Gilman and Knox (1991); and (3) to examine the data for gender differences in the light of recent findings relating to HLA haplotype, and leukaemia susceptibility in males (Taylor et al, 1998).

*Received 5 March 1999*
*Revised 8 November 1999*
*Accepted 8 November 1999*

*Correspondence to:* RJQ McNally

### MATERIALS AND METHODS

### Study subjects

From 1953 to 1973 the MCTR registered all cases of childhood cancer from an area, which included Greater Manchester, Lancashire and parts of Cheshire, Cumbria and Derbyshire (Birch, 1988). The child population was approximately 1 million. Boundary changes reduced the population by about 10% in 1974. Approximately two-thirds of the total regional population live in Greater Manchester.

Cases of ALL and acute non-lymphocytic leukaemia [ANLL], who were diagnosed between 1 January 1954 and 31 December 1985, were analysed (Table 1). For these years, residential addresses at the time of birth as well as at time of diagnosis were consistently available. Eighty children had migrated into the region. In total, 397 of the 798 children for whom addresses at both birth and diagnosis were available had moved within the region. In addition, the locations of cases of lymphoma occurring in the same period were used to provide additional locations of children for some analyses (nearest neighbour analyses).

Ordnance Survey [OS] four-digit grid references were allocated to each case with respect to addresses at time of birth and

**Table 1** Numbers of children by disease group, with birth address and with diagnosis address in the region

| Disease group | Number with birth address in region | Number with diagnosis address in region |
|---|---|---|
| ALL 18–54 months | 292 | 326 |
| ALL 0–4 years | 359 | 399 |
| ALL 5–9 years | 187 | 216 |
| ALL 10–14 years | 101 | 124 |
| ALL 0–14 years | 647 | 739 |
| Leukaemias 0–4 years | 410 | 457 |
| Leukaemias 5–9 years | 230 | 268 |
| Leukaemias 10–14 years | 158 | 183 |
| Leukaemias 0–14 years | 798 | 908 |
| ANLL 0–14 years | 151 | 169 |

diagnosis. Grid references were obtained by locating the residential address on OS maps for the period 1954–1973, and were derived from the postcode for cases diagnosed from 1974 onwards.

The following groups were specified a priori for analysis: total leukaemias aged 0–4, 5–9, 10–14 and 0–14 years; ALL aged 0–4, 5–9, 10–14 and 0–14 years; and ANLL aged 0–14 years. The childhood peak of ALL, consisting of cases aged 18–54 months, was also analysed as a separate group. For each of these groups the reference locations for time were dates of birth and diagnosis and for space were either residential address of the case at the time of diagnosis or at the time of birth. All leukaemias aged 0–14 and 0–4 years; and ALL aged 0–14 and 0–4 years, and the childhood peak were specified, a priori, as likely to show clustering.

To adjust for the effects of different population densities, geographical distance was replaced in some analyses by distance to the Nth nearest neighbour, using all locations of all the cases (of leukaemia and lymphoma) in the data set except addresses for the same child at a different time. N was chosen such that the mean distance was 5 km. N was found to be 75 for birth locations and 68 for diagnosis locations. Jacquez (1996) has used a similar concept and some of our results are based on slight variations of his test.

### Prior hypotheses

The aetiological hypotheses selected a priori to underpin this study were that $H_1$ is true (Greaves and Kinlen hypotheses) and $H_2$ is false (Smith hypothesis) where $H_1$: the primary factor influencing geographical heterogeneity of incidence of childhood leukaemia is related to exposures to one or more common infectious agents occurring post-natally and modulating risk of a late-stage genetic event. A further distinction may be made: susceptibility to exposure is highest at (i) specific ages or (ii) specific times before diagnosis.

$H_2$: The primary factor influencing geographical heterogeneity of incidence of childhood leukaemia is in utero exposure to one or more common infectious agents.

There are four possible space–time interactions: (i) between times and places of birth; (ii) between times and places of diagnosis; (iii) between time of diagnosis and place of birth; and (iv) between time of birth and place of diagnosis.

The interpretation of these possible interactions will depend on the extent of migration between birth and diagnosis among the cases. If there were no migration then these reduce to two possibilities: an interaction between proximity of 'place of residence' and either time of diagnosis or time of birth. The first of these means that cases who lived close to one another had their dates of diagnosis close in time and, hence, share the same spatial–temporal environment at diagnosis and at intervals k months before for any positive k. This would be predicted by $H_1$ (ii) if the agent(s) showed spatial and temporal variability (i.e. occurred in epidemics). The second (interaction between proximities of place of residence and time of birth) means that cases who lived close to one another had their dates of birth close in time and, hence, shared the same spatial–temporal environment at birth, and at 'ages' k months for any k > - 9. This would be predicted by $H_1$ (i) and $H_2$ if the agent(s) generated epidemics. Since, for any pair of children, k cannot exceed the age at diagnosis of either, a stronger signal is predicted from $H_2$ than $H_1$ (i). We note that if $H_1$ is true but neither $H_1$ (i) nor $H_1$ (ii) hold approximately (i.e. the latent period following exposure is variable but susceptibility is not concentrated at particular ages) then neither space–time interactions between residence and time of diagnosis/birth would be predicted even if the agent(s) generated epidemics. Since over half of the children moved between birth and diagnosis, we believe that migration will have some effect, and there will be differences in the space–time clustering effects that are dependent on spatial definitions.

Given that migration is an important factor in the data, the finding of space–time interactions as in (i)–(iv) above would have different interpretations. An interaction between times and places of birth only would indicate an epidemic process affecting fetuses, or newborns, such as an aetiological exposure around the place of birth, prenatally or shortly after the time of birth. It would also indicate that the disease has a variable latent period. An interaction between times and places of diagnosis would indicate a later effect which may be explained by an aetiological exposure around the place of diagnosis and close to the time of diagnosis. It would also indicate that the disease has a short latent period following this exposure. An interaction between birth-addresses and times of diagnosis in the absence of an interaction between times and places of birth would be explained by an aetiological exposure at a variable time after birth, with a fairly constant latent period. An interaction between birth-times and diagnosis places would be explained by an aetiological exposure around the place of diagnosis, with a short latent period and which affects only those in a very narrow age-band. This is not plausible in light of previous results.

Thus, both (ii) and (iii) would be consistent with the Greaves and Kinlen hypotheses together with additional criteria in that a post-natal exposure, migration and population mixing may contribute to these space–time interactions. (i) addresses the Smith hypothesis of a prenatal exposure, using the approach suggested by Gilman and Knox (1991), but is also consistent with variant $H_1$ (i) of the Greaves and Kinlen hypotheses, whilst (iv) is not consistent with the aims and hypotheses and is therefore not considered further.

Since we do not know the actual dates of 'onset', the dates of diagnosis and birth are effectively proxies for this unknown date. Thus proximity of times of birth would correspond to cases having onset at similar ages, whereas closeness of times of diagnosis would correspond to cases diagnosed after a similar latent period.

 © 2000 Cancer Research Campaign

**Table 2** Results of Knox space–time tests for times of diagnosis (Observed space–time pairs[a], strength[b], *P*-value[c])

| | Location at birth | | Location at diagnosis | |
|---|---|---|---|---|
| **Disease group** | **Geographical distance[d]** | **NN threshold[e]** | **Geographical distance[d]** | **NN threshold[e]** |
| ALL 0–4 years | O = 268<br>S = 15.9%<br>(*P* = 0.01) | O = 246<br>S = 13.0%<br>(*P* = 0.03) | O = 265<br>S = 10.4%<br>(*P* = 0.06) | O = 236<br>S = –1.0%<br>N/A |
| ALL 18–54 months | O = 180<br>S = 15.4%<br>(*P* = 0.03) | O = 169<br>S = 14.5%<br>(*P* = 0.045) | O = 175<br>S = 8.0%<br>(*P* = 0.16) | O = 156<br>S = –3.0%<br>N/A |
| ALL 0–14 years | O = 794<br>S = 5.1%<br>(*P* = 0.08) | O = 737<br>S = 6.6%<br>(*P* = 0.044) | O = 875<br>S = 5.0%<br>(*P* = 0.08) | O = 836<br>S = 3.7%<br>(*P* = 0.15) |
| Leukaemias 0–4 years | O = 350<br>S = 14.7%<br>(*P* = 0.006) | O = 306<br>S = 9.5%<br>(*P* = 0.06) | O = 346<br>S = 10.6%<br>(*P* = 0.034) | O = 308<br>S = 0.3%<br>(*P* = 0.49) |
| Leukaemias 0–14 years | O = 1253<br>S = 6.8%<br>(*P* = 0.01) | O = 1098<br>S = 5.2%<br>(*P* = 0.048) | O = 1350<br>S = 7.0%<br>(*P* = 0.007) | O = 1254<br>S = 4.0%<br>(*P* = 0.08) |

[a]Cases are close in time if dates of diagnosis differ by less than 1 year. [b]Strength (S) = (Observed – Expected)/Expected × 100 counts of pairs which are close in time and space. [c]1-sided *P*-value derived from the Poisson distribution. [d]When using geographical distance cases are close in space if their locations are < 5 km apart. [e]When using nearest neighbour (NN) thresholds cases are close in space if the locations of one (or both) was nearer than the other's 75th (68th for addresses at diagnosis) NN in the total data set.

### Knox tests

Space–time (Knox, 1964) tests were applied to the data with thresholds fixed, a priori, as: close in space, less than 5 km and close in time, less than 1 year apart. These limits are somewhat arbitrary, but this problem is overcome by using the K-function method. Where the observed number of pairs which are both close in time and close in space [O], is greater than the expected number [E] this indicates a tendency for pairs of cases which are close in space to have similar times and vice versa. One-sided tests were used to detect a significant interaction. To adjust for different levels of population density, the tests were repeated replacing geographical distance thresholds by the maximum of the distance to the Nth nearest neighbour (N = 75 or 68, see above) of each of the pair.

### Application of K-functions

Two problems are apparent with the Knox test. First, boundary problems may be important since it can be impossible or less probable for some cases to be close in one dimension to other cases. The second problem concerns the arbitrariness of the thresholds chosen, which often results in multiple testing. A simplification, avoiding adjustment for boundary conditions, of a second order procedure based on K-functions (Diggle et al, 1995) is used in the present analyses to overcome the problem of multiple testing. Nearest neighbour approaches were also used as described above in relation to classical Knox tests.

### Histogram analyses

Histogram analyses were performed, as a secondary interpretative analysis, using a modified procedure of Cuzick and Edwards (1990). This procedure enables the identification of cases having unusually large numbers of space–time partners, and therefore cases, influential in the generation of positive results.

**Table 3** Results of Knox space–time tests for times of birth. (Observed space–time pairs[a], strength[b], *P*-value[c])

| | Location at birth | |
|---|---|---|
| **Disease group** | **Geographical distance[d]** | **NN threshold[e]** |
| ALL 0–4 years | O = 232<br>S = 3.7%<br>(*P* = 0.30) | O = 214<br>S = 1.7%<br>(*P* = 0.41) |
| ALL 18–54 months | O = 161<br>S = 5.4%<br>(*P* = 0.26) | O = 149<br>S = 3.1%<br>(*P* = 0.37) |
| ALL 0–14 years | O = 681<br>S = –1.1%<br>N/A | O = 611<br>S = –3.0%<br>N/A |
| Leukaemias 0–4 years | O = 307<br>S = 3.6%<br>(*P* = 0.28) | O = 272<br>S = 0.1%<br>(*P* = 0.50) |
| Leukaemias 0–14 years | O = 1049<br>S = –0.7%<br>N/A | O = 916<br>S = –2.5%<br>N/A |

[a]Cases are close in time if dates of birth differ by less than 1 year. [b]Strength (S) = (Observed − Expected)/Expected × 100 counts of pairs which are close in time and space. [c]1-sided *P*-value derived from the Poisson distribution. [d]When using geographical distance cases are close in space if their locations are < 5 km apart. [e]When using nearest neighbour (NN) thresholds cases are close in space if the locations of one (or both) was nearer than the other's 75th NN in the total data set.

### Analyses by gender of case

Analyses of MCTR data show a higher incidence of c-ALL in boys than in girls aged 1–4 years but not at older ages. Rates per million person-years in males [M] and females [F] aged 1–4 years were, M = 50.9 F = 38.3; in those aged 5–9 were M = 18.1 F = 16.0 and aged 10–14, M = 9.4 F = 9.5 respectively (McNally and Birch, in preparation). This suggests that there may be gender differences in

© 2000 Cancer Research Campaign 

**Table 4** Results of the K-function analysis for space–time[a] clustering by time of diagnosis (Observed integral[b], *P*-value[c])

| Disease group | Location at birth | | Location at diagnosis | |
|---|---|---|---|---|
| | Geographical distance[d] | NN threshold[e] | Geographical distance[d] | NN threshold[e] |
| ALL 0–4 years | 36.14 ($P$ = 0.01) | 38.79 ($P$ = 0.02) | 19.76 ($P$ = 0.07) | 10.6 ($P$ = 0.25) |
| ALL 18–54 months | 21.09 ($P$ = 0.07) | 26.42 ($P$ = 0.07) | 3.73 ($P$ = 0.35) | –1.49 ($P$ = 0.50) |
| ALL 0–14 years | 28.66 ($P$ = 0.02) | 38.09 ($P$ = 0.015) | 24.96 ($P$ = 0.04) | 34.28 ($P$ = 0.04) |
| Leukaemias 0–4 years | 50.28 ($P$ < 0.001) | 33.4 ($P$ = 0.025) | 32.87 ($P$ = 0.007) | 16.88 ($P$ = 0.16) |
| Leukaemias 0–14 years | 51.52 ($P$ = 0.001) | 34.51 ($P$ = 0.02) | 49.18 ($P$ = 0.002) | 48.77 ($P$ = 0.005) |

[a]Cases are close in time if dates of diagnosis differ by < t where t is in the range 1 month–15 months. [b]$\int R(s,t)dsdt$, where $R(s,t) = [K(s,t) – K1(s)K2(t)]/\sqrt{[K1(s)K2(t)]}$, K(s,t) = proportion of pairs of cases whose distances apart are ≤ t in time and ≤ s in space, K1(s) = proportion of pairs whose distance apart is ≤ s, and K2(t) = proportion of pairs whose distance apart in time is ≤ t. [c]*P*-value obtained by simulation (999 runs) with dates of diagnosis randomly re-allocated to the cases in the analysis. [d]Cases are close in space if distances between their locations differ by < s where s is in the range 0.5 km–7.5 km. [e]Cases are close in space if either is within the distance to the *n*th nearest neighbour of the other (in the total data set) where *n* is in the range 71–85 (birth locations) and 63–77 (diagnosis locations).

susceptibility or latent period of onset which are age-dependent. We therefore examined the proportions of male and female cases of ALL aged 0–4 years involved in space–time pairs. This was also examined by applying the $\chi^2$ test for the dichotomy, in one pair/in more than one pair, by sex. The significance of differences in involvement of males and females in space–time pairs was assessed using Mann–Whitney tests.

### RESULTS

### Results of Knox test

The results of these tests based on time of diagnosis for the groups specified above as most likely to display clustering are shown in Table 2. Results with place of birth as location in space give $P < 0.1$ for every group and most results are statistically significant. The strength of the clustering is much greater when younger age groups are analysed alone but this is not always reflected in statistical significance since this also depends on the number of cases available for analysis. By contrast, the evidence of clustering is much weaker if location in space is taken as residence at diagnosis. This is especially evident for the nearest neighbour (NN) analysis. These results are indicative of strong space–time clustering involving all cases, but focusing particularly on younger (0–4 years) cases and do not represent an artefact of varying population density. The evidence, based on the contrast between results for location in space as birth address or diagnosis address points to cases being close together in space at some relatively constant, but probably fairly long, time before their diagnosis. The results based on time and place of birth displayed no evidence of space–time interaction (Table 3). Similarly, other age or diagnostic groups showed little propensity for clustering.

### Results of K-function tests

These tests allow consideration of a range of values of space and time thresholds and also adjusts (via simulation) for edge effects.

**Table 5** Results of the K-function analysis for space–time[a] clustering by time of birth (Observed integral[b], *P*-value[c])

| Disease group | Location at birth | |
|---|---|---|
| | Geographical distance[d] | NN threshold[e] |
| ALL 0–4 years | 8.43 ($P$ = 0.25) | 0.02 ($P$ = 0.51) |
| ALL 18–54 months | 8.70 ($P$ = 0.25) | 3.2 ($P$ = 0.40) |
| ALL 0–14 years | –13.8 ($P$ = 0.82) | –11.72 ($P$ = 0.75) |
| Leukaemias 0–4 years | 7.99 ($P$ = 0.28) | –4.6 ($P$ = 0.63) |
| Leukaemias 0–14 years | –13.7 ($P$ = 0.79) | –19.8 ($P$ = 0.87) |

[a]Cases are close in time if dates of birth differ by < t where t is in the range 1 month – 15 months. [b]$\int R(s,t)dsdt$ – see Table 4 for definitions. [c]*P*-value obtained by simulation (999 runs) with dates of diagnosis randomly re-allocated to the cases in the analysis. [d]Cases are close in space if distances between their locations differ by < s where s is in the range 0.5 km–7.5 km. [e]Cases are close in space if either is within the distance to the *n*th nearest neighbour of the other (in the total data set) where *n* is in the range 71–85 (birth locations).

The results, shown in Table 4, are largely confirmatory of the simple Knox analyses. It is interesting to note, however, that the location at diagnosis analyses are mostly positive for geographical distance although not for NN threshold. An important exception to this is the sub-group for childhood peak ALL for which the data very strongly support the importance of spatial proximity of residences at birth. For most diagnostic/age combinations of interest, clustering is strongest for small thresholds for the location at birth analyses. Again, all other data sets yielded little evidence of clustering (Table 5 gives the place and time of birth results).

 *© 2000 Cancer Research Campaign*

**Table 6** Number of space–time pairs in which individual cases occur by sex of cases

| | Sex | |
|---|---|---|
| | M | F |
| In 1 pair | 43 | 51 |
| In > 1 pair | 86 | 55 |

$\chi^2$, $P = 0.03$.

### Results of histogram tests

The key age/diagnosis groups were inspected for evidence that some cases had unusually large numbers of space–time partners. The Monte-Carlo $P$-values for Pearson's $\chi^2$ statistics indicated non-random distribution for total leukaemias and ALL 0–4 years using both place of residence at birth and diagnosis but not for ALL 18–54 months or all leukaemias 0–4 years.

The cases involved for ALL 0–4 years and total leukaemias were identified. Examination of the 0- to 4-year-old ALLs involved in space–time interactions identified 28 cases who had large numbers of space–time partners. Of the 28, there was a focus on births from 1968 to 1971, and diagnosis from 1972 to 1973. Nine of the 28 cases belonged to a single cluster diagnosed in this period. Exclusion of children diagnosed in 1972–1973 from the data set removed much of the evidence of clustering for young cases, e.g. for ALL aged 0–4 years at diagnosis based on location at birth and time of diagnosis O = 268, E = 231.3 ($P = 0.01$) but when 1972/3 cases are excluded O becomes 210 and E becomes 190.1 ($P = 0.08$).

For all leukaemias aged 0–14 years, the years of diagnosis 1959–1962 were particularly influential and excluding them from the data set removed most of the evidence of clustering in this group, e.g. based on location at diagnosis and time at diagnosis O = 1350, E = 1261.34 ($P = 0.007$) and with 1959–1962 excluded O = 1070, E = 1034.79 ($P = 0.14$).

Thus space–time clustering, although evident in a global analysis of all the data, does not appear to be a generalized phenomenon but rather something which occurs with high intensity in relatively few specific situations.

### Results of analyses of space–time pairs by gender

There were 235 cases in space–time pairs with rather more males than females compared with the overall data (M = 129, F = 106, $0.05 < P < 0.1$). The male cases tended to occur in more pairs (i.e. had more space–time partners than females) (Mann–Whitney test $P = 0.07$). There was a significant excess of males in more than one pair ($P = 0.03$) (Table 6).

Further examination of these space–time pairs revealed a striking female excess in cases which themselves occur in just one pair but are partnered with cases present in multiple pairs (M = 10, F = 28). It could be predicted that such pairs would arise at the beginning (if the singleton case were diagnosed earliest) or the end (if the singleton case were diagnosed latest in the pair) of the clustering in that particular area. Thus the gender of the singleton would be very strongly associated with time. In the data it was seen that singleton males were normally diagnosed earliest in their pair and singleton females latest (Mann–Whitney test for time between two diagnoses by gender of singleton, $P = 0.0004$). These observations could support a hypothesis of a shorter latent period for males and overall for this sub-set (ALL 0–4 years) these results might be considered to be consistent with gender differences in disease susceptibility.

### DISCUSSION

A national study of cases of childhood leukaemia diagnosed during the years 1966–1983 found evidence for space–time clustering, particularly in cases diagnosed under 5 years of age (Gilman and Knox, 1991). This data set included a subset of the cases included in the present analyses (those diagnosed from 1966 to 1983). The authors commented that the results, based on place and date of diagnosis, may be a secondary statistical phenomenon which actually reflected events, important in aetiology, which had occurred prenatally. They observed that a better way to distinguish between hypotheses relating to time of onset of disease or alternatively prenatal events would be to analyse data simultaneously for clustering of dates and places of birth and disease onset. The present analyses address these points and all methods showed highly significant evidence of space–time clustering in our total leukaemia data set (ALL and ANLL aged 0–14 years). This was particularly evident for all leukaemias diagnosed under 5 years, ALL under 5 years and ALL diagnosed during the childhood peak (18 months to 54 months) consistent with our prior hypotheses. There was no evidence of clustering among cases of ANLL analysed alone.

There was no evidence for clustering based on place and time of birth but results based on place of birth/time of diagnosis and place of diagnosis/time of diagnosis both produced significant evidence of clustering. Effectively these results resolve the issue raised by Gilman and Knox (1991) as to whether clustering of cases on place and date of diagnosis actually reflect prenatal events. Our results are not consistent with Smith's hypothesis of prenatal infectious exposure insofar as this relates to an agent or agents which generates epidemics.

The results of analyses based on place of birth and time of diagnosis for the sub-groups ALL aged 0–4, childhood peak ALL and all leukaemias aged 0–14 produced very strong evidence of space–time clustering. The results are consistent with a hypothesis relating to location at some time before the date of diagnosis. The results here suggest a shared exposure followed by a fixed latent period which is relatively long since location at birth is more meaningful than location at diagnosis. When the age differences of the pairs of cases involved in space–time adjacencies were examined, these were usually small and did not differ from those involved in time (of diagnosis) only adjacencies. For 0- to 4-year-old children 85% of those diagnosed within 1 year of each other had dates of birth within 2 years of each other, and for 0- to 14-year-old children 40% had dates of birth within 3 years. Therefore children involved in space–time interactions might be expected to have had contact with the same social communities. Furthermore the clustering appeared to occur in limited geographical areas over rather short time periods and was not a generalized feature throughout the data set.

All these observations are consistent with an infectious aetiology for childhood leukaemia with either or both the mechanisms proposed by Greaves and Kinlen (Greaves, 1988; Kinlen, 1995) operating. Thus the detected space–time clusters would represent mini-epidemics of the disease. Space–time clustering was particularly evident among children diagnosed under 5 years of age and

© 2000 Cancer Research Campaign during the childhood peak (18–54 months). This might suggest that the cases involved are predominantly c-ALL. Greaves' hypothesis applies specifically to c-ALL.

In a recent study of childhood c-ALL, we reported an increased frequency of specific alleles at the DPB1 and DQB1 loci compared with controls. A further analysis of the DQB1 association has shown this to involve a specific DQA1-DQB1 haplotype consisting of DQA1*0101/*0104 DQB1*0501 (Taylor et al, 1998). These alleles are both common in the population and tightly linked to each other, consistent with a role in conferring a selective immunological benefit. Unexpectedly the association was only found in males with c-ALL. In the context of an infectious aetiology in c-ALL, this might be consistent with a stronger immune response to infection accruing from a specific HLA haplotype more strongly in males than females, as a means of counteracting the effects of X-linked hemizygosity for immune-associated genes in males compared with females. The results showing gender differences, in particular an excess of males involved in space–time pairs, add weight to the concept of differential susceptibility in males and females.

The EUROCLUS project found significant evidence of spatial clustering among all childhood leukaemias which could not be attributed to any specific age group or cell type of leukaemia (Alexander et al, 1998*b*). In these data, when spatial–temporal patterns were examined in areas which showed clustering, there was significant evidence of space–time interactions between cases which included different age and diagnostic sub-groups (Alexander et al, 1998*a*). Results were interpreted as supporting an infectious origin. The highly significant results obtained in the present analyses for the all leukaemia data set suggest that, although cases of ANLL do not cluster significantly with each other, they may contribute to the overall pattern of clustering with more than one mechanism involving infections operating.

There are certain limitations to the methodology that has been used in this study. Mantel (1967) pointed out that the Knox (1964) test is biased if there are population shifts during the time period, such as when the population grows or declines with different percentages in different areas of the study region. Our paper provides a description of the space–time clustering apparent in the data, whether artefactual or real. However, it is believed that variations in population growth are unimportant in the present data set.

In summary, we have found strong evidence of space–time clustering among cases of childhood leukaemia, particularly ALL and cases diagnosed at early ages. Evidence for clustering was strongest when residential address at time of birth was used as the reference point in space. The concentration of clustering among young cases suggests the importance of c-ALL in the space–time interactions. However, there is a suggestion of a complex pattern with possible interactions between diagnostic sub-groups. The results are consistent with a role for infection in the aetiology of childhood leukaemia but the complex pattern of clustering suggests that more than one mechanism may be operating. Future studies should consider diagnostic sub-groups defined by biological markers separately, or in appropriate combinations to test hypotheses relating to common underlying aetiological factors.

### ACKNOWLEDGEMENTS

The Manchester Children's Tumour Registry is supported by the Cancer Research Campaign. Jillian M Birch is Cancer Research Campaign Professorial Research Fellow in Paediatric Oncology and Osborn B Eden is Cancer Research Campaign Professor of Paediatric Oncology at the University of Manchester.

### REFERENCES

Alexander FE, Boyle P, Carli PM, Coebergh JW, Draper GJ, Ekbom A, Levi F, McKinney PA, McWhirter W, Magnani C, Michaelis J, Olsen JH, Peris-Bonet R, Petridou E, Pukkala E and Vatten L, on behalf of the EUROCLUS project (1998*a*) Spatial temporal patterns in childhood leukaemia: further evidence for an infectious origin. *Br J Cancer* **77**: 812–817

Alexander FE, Boyle P, Carli PM, Coebergh JW, Draper GJ, Ekbom A, Levi F, McKinney PA, McWhirter W, Michaelis J, Peris-Bonet R, Petridou E, Pompe-Kirn V, Plisko I, Pukkala E, Rahu M, Storm H, Terracini B, Vatten L and Wray N, on behalf of the EUROCLUS project (1998*b*) Spatial clustering of childhood leukaemia: summary results from the EUROCLUS project. *Br J Cancer* **77**: 818–824

Birch JM (1988) Manchester Children's Tumour Registry 1954–1970 and 1971–1983. In: *International Incidence of Childhood Cancer*, Parkin DM, Stiller CA, Draper GJ, Bieber CA, Terracini B and Young JL (eds), pp. 299–304. IARC Scientific Publications no. 87. IARC: Lyon

Cuzick J and Edwards R (1990) Spatial clustering for inhomogeneous populations (with discussion). *J R Stat Soc B* **52**: 73–104

Diggle PJ, Chetwynd AG, Haggkvist R and Morris SE (1995) Second-order analysis of space-time clustering. *Stat Methods Med Res* **4**: 124–136

Gilman EA and Knox G (1991) Temporal–spatial distribution of childhood leukaemias and non-Hodgkin lymphomas in Great Britain. In: *The Geographical Epidemiology of Childhood Leukaemia and Non-Hodgkin Lymphomas in Great Britain, 1966–1983*, Draper GJ (ed), pp. 77–99. Studies on Medical and Population Subjects no. 53. HMSO: London

Greaves MF (1988) Speculations on the cause of childhood acute lymphoblastic leukemia. *Leukemia* **2**: 120–125

Jacquez GM (1996) A *k* nearest neighbour test for space–time interaction. *Stat Med* **15**: 1935–1949

Kinlen L (1988) Evidence for an infective cause of childhood leukaemia: comparison of a Scottish New Town with nuclear reprocessing sites in Britain. *Lancet* **ii**: 1323–1327

Kinlen L (1995) Epidemiological evidence for an infective basis in childhood leukaemia. *Br J Cancer* **71**: 1–5

Knox EG (1964) The detection of space–time interactions. *Applied Stats* **13**: 25–29

Mantel N (1967) The detection of disease clustering and a generalized regression approach. *Cancer Res* **27**: 209–220

Smith M (1997) Considerations on a possible viral etiology for B-precursor acute lymphoblastic leukemia of childhood. *J Immunother* **20**: 89–100

Taylor GM, Dearden S, Payne N, Ayres M, Gokhale DA, Birch JM, Blair V, Stevens RF, Will AM and Eden OB (1998) Evidence that an HLA-DQA1-DQB1 haplotype influences susceptibility to childhood common acute lymphoblastic leukaemia in boys provides further support for an infection-related aetiology. *Br J Cancer* **78**: 561–565

Wiemels JL, Cazzaniga G, Daniotti M, Eden OB, Addison GM, Masera G, Saha V, Biondi A and Greaves MF (1999) Prenatal origin of acute lymphoblastic leukaemia in children. *Lancet* **354**: 1499–1503

*© 2000 Cancer Research Campaign*
